# High quality draft genome sequence of *Segniliparus rugosus* CDC 945^T^= (ATCC BAA-974^T^)

**DOI:** 10.4056/sigs.2255041

**Published:** 2011-12-30

**Authors:** Ashlee M. Earl, Christopher A. Desjardins, Michael G. Fitzgerald, Harindra M. Arachchi, Qiandong Zeng, Teena Mehta, Allison Griggs, Bruce W. Birren, Nadege C. Toney, Janice Carr, James Posey, W. Ray Butler

**Affiliations:** 1 Genome Sequencing and Analysis Program, Broad Institute of MIT and Harvard, 7 Cambridge Center, Cambridge, MA, USA; 2 Division of Tuberculosis Elimination, Laboratory Branch, Centers for Disease Control and Prevention, Atlanta, Georgia, USA; 3 Division of Healthcare Quality Promotion, Clinical and Environmental Microbiology Branch, Centers for Disease Control and Prevention, Atlanta, Georgia, USA

**Keywords:** *Segniliparaceae,*genome sequencing, Human Microbiome Project

## Abstract

*Segniliparus rugosus* represents one of two species in the genus *Segniliparus*, the sole genus in the family *Segniliparaceae*. A unique and interesting feature of this family is the presence of extremely long carbon-chain length mycolic acids bound in the cell wall. *S. rugosus* is also a medically important species because it is an opportunistic pathogen associated with mammalian lung disease. This report represents the second species in the genus to have its genome sequenced. The 3,567,567 bp long genome with 3,516 protein-coding and 49 RNA genes is part of the NIH Roadmap for Medical Research, Human Microbiome Project.

## Introduction

Strain CDC 945^T^ (= ATCC BAA-974^T^ = CIP 10838^T^ = DSM 45345 = CCUG 50838^T^ = JCM 13579^T^) is the type strain of the species *Segniliparus rugosus* in the *Segniliparaceae* family [1]. The genus name was created to acknowledge the presence of novel long carbon-chain fatty acids (mycolic acids) detected using the *Mycobacterium* species identification method with high performance liquid chromatography (HPLC) [2]. The name was formed from the Latin adjective ‘*segnis’,* meaning ‘slow’ and combined with the Greek adjective ‘*liparos’* for ‘fatty’, to indicate the ‘one with slow fats’. The name relates to the late elution of the apolar, alpha-mycolic acids (fatty acids) during HPLC analysis [1]. The specific epithet for the taxon name is from the Latin adjective ‘*rugosus*’, referring to the formation of wrinkled, rough colony morphology [1]. The type strain of *S. rugosus*, CDC 945^T^, was isolated from a human sputum specimen collected in Alabama, USA [1]. *S. rugosus* has been isolated from multiple patients with cystic fibrosis in the U.S. and Australia and appears to be a respiratory opportunistic pathogen [3,4]. A recent isolation from a ~1 year old sea lion showing third-stage malnutrition with a 30% loss of body weight, moderate bradycardia and severe hypothermia, suggests a possible aquatic or marine niche for the species [5]. The only other validly named species of the genus is *Segniliparus rotundus* (CDC 1076^T^), which is the type strain of this species. *S. rotundus*. CDC 1076 shares 98.9% 16S rRNA sequence identity with *S. rugosus* CDC 945^T^, although the DNA-DNA hybridization is less than 28% [1]. The complete genome of *S. rotundus* was recently reported and has 3,157,527 bp with 3,081 protein-coding and 52 RNA genes [6]. Here we present a summary classification and a set of features for *S. rugosus* CDC 945^T^, together with the description of the high quality draft genomic sequencing and annotation.

## Classification and features

**Figure1:**
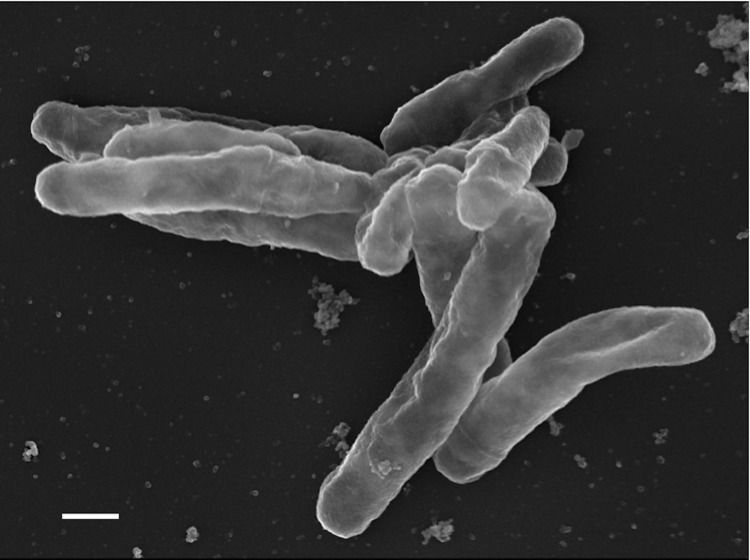
Scanning electron micrograph of *S. rugosus* CDC 945^T^. The scale bar is 667nm.

The cells of CDC 945^T^ are irregular rods ranging in length and width from 0.55-0.90 µm by 1.9-4.5 µm (Table 1 and Figure 1). Colonies are wrinkled, rough and form in less than 7 days on 7H10 and 7H11 agar at an optimal temperature of 33^o^C [1]. CDC 945^T^ is aerobic, non-motile, asporogenous, and stains bright red with acid alcohol stain [1]. It is mesophilic and demonstrates a temperature range for growth between 22 and 42 ^o^C [1]. Colonies grown for < 4 weeks are non-pigmented, nonphotochromogenic and do not produce a diagnostic odor [1]. They do not produce aerial mycelium, spores or demonstrate true branching. Young colonies are creamy and smear easily when disturbed. Cell growth at ~4 weeks on Löwenstein-Jensen (LJ) medium produces a diffusible pink color in the agar at the leading edges of mature growth. Aged colonies on LJ develop a light buff pigment and demonstrate ‘greening’ from uptake of malachite green [1]. CDC 945^T^ is weakly positive for arylsulfatase at 7 days but is strongly positive in 14 days. No growth on MacConkey agar not containing crystal violet. CDC 945^T^ grows in the presence of 5% sodium chloride at 7 days, in lysozyme at 21 days. Positive for iron uptake, nitrate reduction, tellurite reduction and tween opacity. Negative for tween hydrolysis [1].

**Table1:** Classification and general features of *S. rugosus* CDC 945^T^ according to the MIGS recommendations [25].

**MIGS ID**	**Property**	**Term**	**Evidence code**
		Domain *Bacteria*	TAS [26]
		Phylum *Actinobacteria*	TAS [27]
		Class *Actinobacteria*	TAS [28]
		Subclass *Actinobacteridae*	TAS [28]
		Order *Actinomycetales*	TAS [28,29]
	Current classification	Suborder *Corynebacterineae*	TAS [28,29]
		Family *Segniliparaceae*	TAS [1,29]
		Genus *Segniliparus*	TAS [1]
		Species *Segniliparus rugosus*	TAS [1]
		Type strain CDC 945	TAS [1]
	Gram stain	not reported	
	Cell shape	rods, irregular	TAS [1]
	Motility	nonmotile	TAS [1]
	Sporulation	non-sporulating	TAS [1]
	Temperature range	mesophile, 22-42 ^o^C	TAS [1]
	Optimum temperature	33^o^C	TAS [1]
	Salinity	unknown	
MIGS-22	Oxygen requirement	aerobic	TAS [1]
	Carbon source	D-glucose, glycerol, maltose, mannitol, D-sorbitol and trehalose	TAS [1]
	Energy source	chemoorganotroph	TAS [1]
MIGS-6	Habitat	environmental water suggested	TAS [5]
MIGS-15	Biotic relationship	likely free-living	NAS [5]
MIGS-14	Pathogenicity	opportunistic pathogen	TAS [1,3]
	Biosafety level	2	TAS [1,3,30]
	Isolation	sputum, human	TAS [1,3]
MIGS-4	Geographic location	Alabama, USA	TAS [1]
MIGS-4.1	Latitude	not reported	
MIGS-4.2	Longitude	not reported	
MIGS-4.3	Depth	not reported	
MIGS-4.4	Altitude	not reported	
MIGS-5	Sample collection time	1998	TAS [1]

Evidence codes- TAS: Traceable Author Statement (i.e., a direct report exists in the literature); NAS Non-traceable Author Statement (i.e., not directly observed for the living, isolated sample, but based on a generally accepted property for the species, or anecdotal evidence). These evidence codes are from the Gene Ontology project [31].

Results with the API CORYNE test kit shows CDC 945^T^ is positive for β-glucosidase, and pyrazinamidase activities and negative for alkaline phosphatase, β-galactosidase, β-glucuronidase, α-glucosidase, *N*-acetyl-β-glucosaminidase and pyrrolidonyl arylamidase activity at 33^o^C [1]. It is susceptible to imipenem 4ug/ml, moxifloxacin 0.5 μg/ml, and trimethoprim-sulfamethoxazole < 4.8 ug/ml, intermediate to cefoxitan 64 ug/ml and resistant to amikacin >128 ug/ml, clarithromycin 32 ug/ml, ciprofloxacin 16 ug/ml, ethambutol >16 ug/ml and tobramycin >64 ug/ml. [1,3]. Strain CDC 945^T^ uses D-glucose, glycerol, maltose, mannitol, D-sorbitol and trehalose as sole carbon sources with the production of acid. No growth on adonitol, L-arabinose, cellobiose, citrate, dulcitol, i-erythriol, galactose, i-*myo*-inositol, lactose, mannose, melibiose, raffinose, L-rhamnose, salicin or sodium citrate [1]. The strain hydrolyzes urea but not acetamide adenine, casein, aesculin, hypoxanthine, tyrosine or xanthine [1].

### Chemotaxonomy

The cell wall of strain CDC 945^T^ contains mycolic acids and meso-diaminopimelic acid [1]. The mycolic acid pattern developed with HPLC is a double cluster of peaks emerging at 7.24 min and the last peak group is unresolved and elutes slightly before the 110 carbon chain length, high molecular weight internal standard [1,2]. Thin layer chromatography confirms 2 groups of apolar, α- and α'-alpha-mycolic acids lacking oxygen function, other than the hydroxyl group [1]. The HPLC and TLC results indicate that this strain produces a unique homologous subclass of long, alpha-mycolic acids with additional 90 to 110 carbons [1]. The fatty acid profile by gas-liquid chromatography is C_10:0_ (8.65%), C_12:0_ (1.33%), C_14:0_ (8.49%), C_16:0_ (18.34%), C_18:1ω9c_ (8.93%), C_18:0__10-methyl_ (tuberculostearic acid, 21.62%), and C_20_ (28.51%) [1].

**Figure 2:**
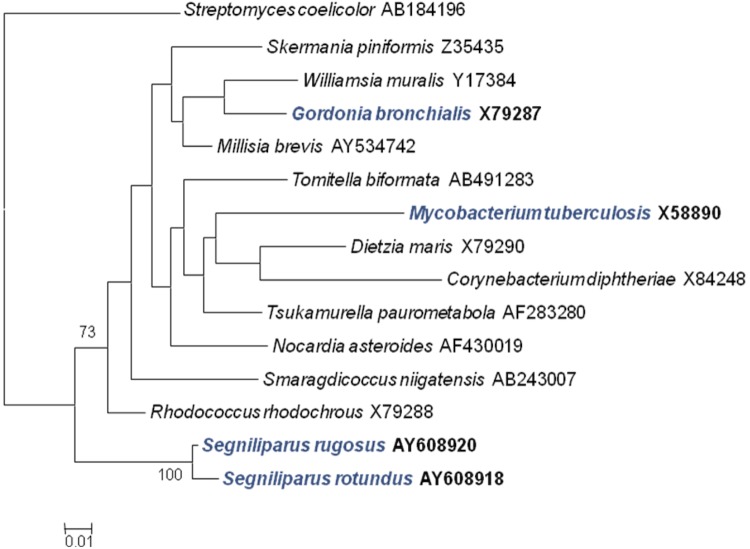
Maximum likelihood phylogenetic tree was generated using PHYML v2.2.4 [7] based on 16S rRNA sequences highlighting the position of *S. rugosus* CDC 945^T^ relative to the other type strains of mycolic acid containing genera in the suborder *Corynebacterineae*. GenBank accession numbers are listed after the name. The tree was inferred from 1,468 bp positions aligned using Clustal W [8] in MEGA v4 [9]. Numbers at the branch nodes are support values from 1,000 bootstrap replicates if equal to or greater than 70%. The scale bar indicates substitutions per site. The tree was rooted with *Streptomyces coelicolor*. Lineages with type strain genome sequencing projects registered in **GOLD** [10] are shown in blue, published genomes in bold.

The phylogenetic association of *Segniliparus* species is shown in Figure 2 in a 16S rRNA based tree. The genus forms a distinct lineage relative to the other mycolic acid containing *Actinobacteria*. The positioning of *Segniliparus* in this phylogenetic analysis is consistent with its positioning in the “All-Species Living Tree Project” LTP release 106, August 2011, which is similarly based on 16S rRNA. [11].

## Genome sequencing and annotation

### Genome project history

This organism was selected for sequencing on the basis of its association with respiratory lung disease and is part of the NIH Roadmap for Medical Research, Human Microbiome Project (HMP) [12]. The HMP presents reference genomes in Genome Online Database (GOLD) [10], the Human Microbiome Project Data Analysis and Coordination Center Project Catalog [13] and the complete high quality draft genome sequence is deposited in GenBank [14]. The Broad Institute performed the sequencing and annotation of this high quality draft genome [15]. A summary of the project is given in Table 2.

**Table 2:** Genome sequencing project information

**MIGS ID**	**Property**	**Term**
MIGS-31	Finishing quality	High Quality Draft
MIGS-28	Libraries used	Two 454 pyrosequence libraries, one standard 0.6kb fragment library and one 2.5kb jump library
MIGS-29	Sequencing platforms	454 Titanium
MIGS-31.2	Sequencing coverage	13×
MIGS-30	Assemblers	Newbler Assembler version 2.3 PostRelease-11/19/2009
MIGS-32	Gene calling method	Glimmer; Metagene; PFAM; BLAST to non-redundant protein database; manual curation
	Genbank ID	ACZI01000000
	Genbank Date of Release	November 10, 2010
	GOLD ID	Gi05259
	NCBI project ID	40685
MIGS-13	Source material identifier	ATCC BAA-974^T^
	Project relevance	Human Microbiome Project

### Growth conditions and DNA isolation

Strain CDC 945^T^ was grown statically in Middlebrook 7H9 medium at 33^o^C until late log. DNA was isolated from whole cells after a chloroform/methanol wash with a disruption solution of guanidine thiocyamate, sarkosyl and mercaptoethanol as described in Mve-Obiang et al. [16]. The purity of DNA was assessed by The Broad Institute using the Quant-iT™ dsDNA Assay High Sensitivity Kit (Invitrogen, Carlsbad, CA) and according to the manufacturer's protocol.

### Genome sequencing and assembly

The genome of *Segniliparus rugosus* ATCC BAA-974 was sequenced using 454 pyrosequence fragment and jump libraries [17]. We assembled the 454 data, consisting of 135,510 fragment reads and 112,271 jump reads, using Newbler Assembler version 2.3 PostRelease-11/19/2009. The assembly is considered High-Quality Draft and consists of 262 contigs arranged in 30 scaffolds with a total size of 3,567,567 bases. The error rate of this draft genome sequence is less than 1 in 10,000 (accuracy of ~ Q40). Average sequence coverage is 13×. Assessment of coverage, GC content, contig BLAST and 16S contig classification were consistent with the species *Segniliparus*

### Genome annotation

Protein-coding genes were predicted using four ORF-finding tools: GeneMark [18], Glimmer3 [19], Metagene [20], and findBlastOrfs (unpublished). This latter tool builds genes by extending whole-genome blast alignments, in-frame, to include start and stop codons. The final set of non-overlapping ORFs was selected from the output of these tools using an in-house gene-caller, which uses dynamic programming to score candidate gene models based on strength of similarity to entries in UniRef90, then selects non-overlapping genes that, combined, have the highest overall score. In cases where predictions overlapped non-coding RNA features (see below), the genes were manually inspected and removed when necessary. Finally, the gene set was reviewed using both the NCBI discrepancy report and the internal Broad annotation metrics. Ribosomal RNAs (rRNAs) were identified with RNAmmer [21]. The tRNA features were identified using tRNAScan [22]. Other non-coding features were identified with RFAM [23]. The gene product names were assigned based on Hmmer equivalogs from TIGRfam and Pfam, and blast hits to KEGG and SwissProt protein sequence databases. This was done using the naming tool “Pidgin” [24].

## Genome properties

**Figure 3:**
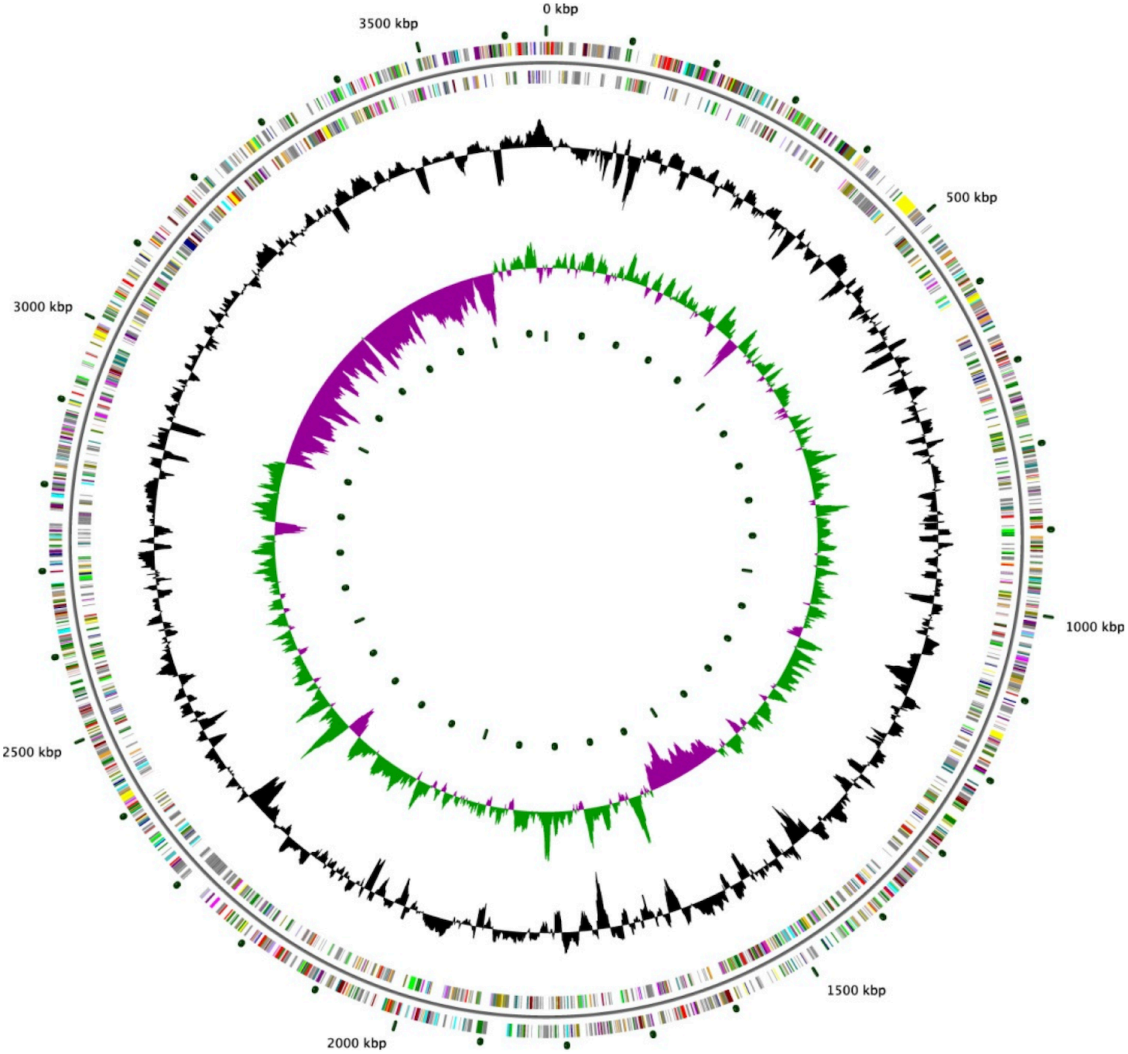
Graphical circular map of the genome. From outside to the center: Genes on forward strand (color by COG categories), Genes on reverse strand (color by COG categories), GC content, GC skew.

This 3,567,567 bp draft genome has high G+C content (Table 3 and Figure 3) and is predicted to encode 3,571 genes, 98% of which are protein coding. Nearly 70% of predicted proteins have a functional prediction and COG functional categories have been assigned to 53% of predicted proteins (Table 4).

**Table 3:** Genome Statistics

**Attribute**	**Value**	**% of Total**
Genome size (bp)	3,647,826	100.00%
DNA coding region (bp)	3,156,492	86.35%
DNA G+C content (bp)	2,484,899	68.12%
Number of replicons	unknown	
Extrachromosomal elements	unknown	
Total genes	3,571	100.0%
tRNA genes	46	1.28%
rRNA genes	3	0.08%
rRNA operons	1	0.03%
CRISPR repeats	0	
Protein-coding genes	3,522	98.62%
Pseudo genes (partial genes)	6 (233)	0.17% (6.52%)
Genes with function prediction	2,486	69.62%
Genes in paralog clusters	194	5.43%
Genes assigned to COGS	1,897	53.12%
Genes assigned Pfam domains	2,451	68.64%
Genes with signal peptides	412	11.54%
Genes with transmembrane helices	590	16.52%

**Table 4:** Number of genes associated with the general COG functional categories

Code	Value	%age	Description
J	121	3.4	Translation, ribosomal structure and biogenesis
A	1	0.0	RNA processing and modification
K	89	2.5	Transcription
L	84	2.4	Replication, recombination and repair
B	0	0.0	Chromatin structure and dynamics
D	18	0.5	Cell cycle control, cell division, chromosome partitioning
Y	0	0.0	Nuclear structure
V	22	0.6	Defense mechanisms
T	51	1.4	Signal transduction mechanisms
M	85	2.4	Cell wall/membrane/envelope biogenesis
N	3	0.1	Cell motility
Z	0	0.0	Cytoskeleton
W	0	0.0	Extracellular structures
U	11	0.3	Intracellular trafficking and secretion, and vesicular transport
O	77	2.2	Posttranslational modification, protein turnover, chaperones
C	140	4.0	Energy production and conversion
G	100	2.8	Carbohydrate transport and metabolism
E	225	6.4	Amino acid transport and metabolism
F	74	2.1	Nucleotide transport and metabolism
H	102	2.9	Coenzyme transport and metabolism
I	120	3.4	Lipid transport and metabolism
P	110	3.1	Inorganic ion transport and metabolism
Q	92	2.6	Secondary metabolites biosynthesis, transport and catabolism
R	246	7.0	General function prediction only
S	126	3.6	Function unknown
-	1625	46.1	Not in COGs
